# Utilizing disease transmission and response capacities to optimize covid-19 control in Malaysia

**DOI:** 10.1186/s12889-024-18890-3

**Published:** 2024-05-28

**Authors:** Sarbhan Singh, Lai Chee Herng, Nuur Hafizah Md. Iderus, Sumarni Mohd Ghazali, Lonny Chen Rong Qi Ahmad, Nur’ain Mohd Ghazali, Mohd Nadzmi Md Nadzri, Asrul Anuar, Mohd Kamarulariffin Kamarudin, Lim Mei Cheng, Kok Keng Tee, Chong Zhuo Lin, Balvinder Singh Gill, Nur Ar Rabiah Binti Ahmad

**Affiliations:** 1 Institute for Medical Research (IMR), National Institutes of Health (NIH), Ministry of Health Malaysia, No.1, Jalan Setia MurniSetia Alam, U13/52, Seksyen Selangor, Malaysia; 2 Department of Medicine, Faculty of Medicine, Universiti Malaya, Kuala Lumpur, Malaysia; 3 Institute for Public Health (IPH), National Institutes of Health (NIH), Ministry of Health Malaysia, Setia Alam, 40170 Malaysia

**Keywords:** COVID-19, Community transmission, Response capacity, Situational level, Public health

## Abstract

**Objectives:**

Public Health Social Measures (PHSM) such as movement restriction movement needed to be adjusted accordingly during the COVID-19 pandemic to ensure low disease transmission alongside adequate health system capacities based on the COVID-19 situational matrix proposed by the World Health Organization (WHO). This paper aims to develop a mechanism to determine the COVID-19 situational matrix to adjust movement restriction intensity for the control of COVID-19 in Malaysia.

**Methods:**

Several epidemiological indicators were selected based on the WHO PHSM interim guidance report and validated individually and in several combinations to estimate the community transmission level (CT) and health system response capacity (RC) variables. Correlation analysis between CT and RC with COVID-19 cases was performed to determine the most appropriate CT and RC variables. Subsequently, the CT and RC variables were combined to form a composite COVID-19 situational matrix (SL). The SL matrix was validated using correlation analysis with COVID-19 case trends. Subsequently, an automated web-based system that generated daily CT, RC, and SL was developed.

**Results:**

CT and RC variables were estimated using case incidence and hospitalization rate; Hospital bed capacity and COVID-19 ICU occupancy respectively. The estimated CT and RC were strongly correlated [*ρ* = 0.806 (95% CI 0.752, 0.848); and *ρ* = 0.814 (95% CI 0.778, 0.839), *p* < 0.001] with the COVID-19 cases. The estimated SL was strongly correlated with COVID-19 cases (*ρ* = 0.845, *p* < 0.001) and responded well to the various COVID-19 case trends during the pandemic. SL changes occurred earlier during the increase of cases but slower during the decrease, indicating a conservative response. The automated web-based system developed produced daily real-time CT, RC, and SL for the COVID-19 pandemic.

**Conclusions:**

The indicators selected and combinations formed were able to generate validated daily CT and RC levels for Malaysia. Subsequently, the CT and RC levels were able to provide accurate and sensitive information for the estimation of SL which provided valuable evidence on the progression of the pandemic and movement restriction adjustment for the control of Malaysia.

## Introduction

The COVID-19 pandemic had resulted in an unprecedented worldwide crisis since it was first discovered late in December 2019 [1, 2]. Globally, the implementation of Public Health and Social Measures (PHSM) during the initial phases of the pandemic was instrumental in slowing the spread of COVID-19, therefore preventing healthcare systems from becoming overwhelmed [3, 4]. These PHSMs include non –pharmaceutical interventions such as movement restrictions, personal protective measures (i.e. hand hygiene, respiratory etiquette, mask-wearing), environmental measures (i.e. cleaning, disinfection, ventilation), and physical distancing [5].

PHSM especially movement restrictions have been proven to be effective in limiting the transmission of COVID-19 across many countries [3]. However, sustained prolonged movement restrictions are not feasible due to their undesirable effects on the socioeconomic health of a nation [3]. While the World Health Organization (WHO) advised that lifting of PHSM (i.e. movement restrictions) prematurely would result in a more severe resurgence of COVID-19 infections, health authorities worldwide need to strike a balance between instituting PHSM to control the pandemic and its negative effects on the nations socioeconomic health [3, 6]. Therefore, countries worldwide currently face a common challenge in determining when to continue, ease or lift PHSM for the control and management of the pandemic [3]. In this regard, researchers have examined pandemic exit strategies adopted by various countries and have identified several prerequisites for adjusting COVID-19 PHSM namely information on infection status, community engagement, adequate public health-system capacity, and border controls [5]. In addition, WHO released interim guidance reports on ‘Considerations for implementing and adjusting PHSM in the context of the pandemic’ in November 2020 and a revision in July 2021 [5]. The fundamental principles provided enable relevant authorities to make evidence-based decisions that are tailored for local situations to optimize the application and adjustments of PHSM in the control of COVID-19 [5].

The intensity of the pandemic has been influenced by several factors related to the disease dynamics and the control measures that were instituted [3]. In Malaysia, the most significant control measure instituted early in the pandemic was the movement restriction measure which was called the Movement Control Order (MCO) to prevent the disease from overwhelming the healthcare system [7]. The institution of the MCO was first implemented on 18 March 2020 and had to take into account the level of movement restriction and its subsequent negative effects on the socioeconomic health of the nation over time to strike a balance in the control of the pandemic [8]. To achieve the best outcomes and to prevent negative socioeconomic effects, the MCO over time should be adjusted accordingly based on various epidemiological, social, and economic parameters [9].

In this paper, we adapted the WHO interim guidance report to develop the daily COVID-19 Situational Level assessment matrix which would provide a mechanism to adjust the level of movement restriction for the control of the COVID-19 pandemic in Malaysia for the two-year pandemic duration from 1 April 2020 to 31 March 2022. This would include firstly selecting and validating the relevant indicators to be used for estimating disease Community Transmission (CT) and Health System Response Capacity (RC) levels. Secondly, the Situational Level assessment matrix will be determined using the validated CT and RC levels. Thirdly, this paper describes the development of a web-based automated surveillance system that enables continuous estimation and access to daily CT, RC, and COVID-19 Situational Level assessment matrix in Malaysia. With the availability of the WHO interim guidance report, it is advised that each country adopt, modify and validate this guidance based on their respective local context to develop a COVID-19 Situational Level assessment matrix. This would be able to provide evidence for making informed decisions when adjusting PHSM for the control and management of the pandemic [5].

This study represents a pioneering effort in addressing the COVID-19 pandemic within Malaysia, filling significant research gaps by focusing on the nation's context, which has been largely overlooked in existing literature discussing the utilization of World Health Organization (WHO) interim guidance for adjusting Public Health and Social Measures (PHSM) during the pandemic [10–12]. Leveraging these WHO guidelines, the research charts new territory in pandemic response strategies tailored specifically to the Malaysian landscape, providing directly applicable insights for the formulation of public health policies and strategies. Notably, the study introduces a novel framework for determining the COVID-19 situational matrix, a dynamic tool designed to optimize the application and adjustments of PHSM. By acknowledging the complex interplay between disease transmission dynamics and the capacity of the health response infrastructure, this innovative approach offers a deeper understanding that can inform more effective and targeted interventions. The development of an automated web-based system to generate daily real time COVID-19 situational matrix serves as an added novelty of this work. Furthermore, at an International level, this would be considered as the first adaptation of WHO guidelines for development the COVID-19 situational matrix for National level COVID-19 response. The study's novelty lies not only in its Malaysian focus but also in its innovative methodology, which paves the way for a more agile and data-driven approach to managing the COVID-19 pandemic in Malaysia, emphasizing the need for such research to develop mechanisms for adjusting PHSM for pandemic control. Hence the need to conduct this study to develop a mechanism to determine the COVID-19 situational matrix to adjust movement restriction intensity for the control of the COVID-19 pandemic in Malaysia.

## Literature review

Understanding the dynamics of disease transmission and the capacity of health systems to respond is crucial for informing effective implementation and adjustments of Public Health and Social Measures (PHSM). Theoretical frameworks, such as the Law of Demand and Supply, contribute to a comprehensive understanding of the interplay between health response capacity and outbreak intensity. For instance, more severe outbreaks necessitate higher health response capacity to manage them [13]. Moreover, the Transmission Dynamics model provides a rationale for considering community transmission as a critical variable when assessing outbreak intensity. This is because higher transmission rates ultimately lead to more severe outbreaks [14]. Furthermore, concepts from epidemiology and health systems research contribute significantly to a comprehensive understanding of the interplay between disease transmission dynamics and health response capacities. These insights guide the development of strategies to optimize adjustments to PHSM [15–19].

The World Health Organization (WHO) has been instrumental in providing guidance on PHSM implementation and adjustments, emphasizing the importance of evidence-based decision-making and context-specific approaches [5]. Studies from various countries have evaluated the effectiveness of PHSM in controlling COVID-19 transmission and identified factors influencing their implementation and adjustments [20–23]. Lessons learned from international experiences offer valuable insights into best practices and challenges in optimizing PHSM adjustments.

Malaysia's response to the COVID-19 pandemic has involved the implementation of various PHSM, including movement restrictions, border controls, and testing strategies [24–26]. Challenges in implementing and adjusting PHSM have included balancing disease control with economic considerations, addressing disparities in healthcare access, and ensuring community compliance with preventive measures [24–27]. Evaluating Malaysia's experiences with PHSM implementation and adjustments can provide valuable lessons for optimizing future strategies.

Innovative methodologies and frameworks have been developed to optimize PHSM adjustments based on disease transmission dynamics and health response capacities. These include dynamic modeling approaches, risk assessment tools, and decision-making frameworks that integrate epidemiological data with socio-economic indicators [28–30]. The WHO interim guidance for adjusting PHSM during the pandemic provides one such framework that integrates several of these methodologies into it [5].

In conclusion, optimizing the application and adjustments of PHSM is critical for controlling COVID-19 in Malaysia and worldwide. Drawing on theoretical frameworks, global perspectives, and specific experiences, provides valuable insights into strategies for utilizing disease transmission intensity and health response capacities to inform decision-making. By addressing gaps in knowledge and practice and exploring novel approaches, researchers and policymakers can enhance pandemic response efforts and mitigate the impact of COVID-19 on public health and society.

## Materials and methods

### Data sources

The data for the study period which covers the two-year duration of the COVID-19 pandemic in Malaysia before entering the endemic phase was from 1 April 2020 to 31 March 2022 [31]. National COVID-19 cases, active cases, deaths, and tests were obtained from the Ministry of Health (MOH), Malaysia's official press statement (available at http://covid-19.moh.gov.my/), and the MOH official Github data repository (available at https://github.com/MoH-Malaysia/covid19-public). National COVID-19 effective reproduction was sourced from the MyR-number web application system [8]. National population data were obtained from the Department of Statistics Malaysia (DOSM)(available at https://www.dosm.gov.my/v1_/).

### Data analysis

Data analysis is described in the following paragraphs.

### Selection and validation of the indicators to estimate the CT and RC levels

#### Community transmission levels (CT)

The intensity of disease transmission was estimated by determining the CT values of the disease. The CT values are derived from selecting the indicators such as hospitalization rate, mortality rate, case incidence, and testing rate and determining their cut-offs. The CT values are categorized into four levels, from low incidence (CT1) to very high incidence (CT4) [5].

- Selection of indicator and defining cuts off.

The epidemiological indicators used to assess the CT level were hospitalization rate, mortality, case incidence, and effective reproduction number (Rt) as shown in Table 1. The WHO standard cut-offs were used to define all the indicators to the respective CT levels (Table 1). All cut-offs were validated during the validation process (described below). Indicators were used individually and in combinations to determine the most appropriate CT values. Case incidence was used as a common indicator for each combination of indicators, therefore resulting in a total of 5 combinations as shown in Appendix 1.

**Table 1:** Indicators to estimate CT and RC levels, in Malaysia

COVID-19 CT levels								
**No**	**Indicators**	**Formula**	**CT 1**	**CT 2**		**CT3**		**CT4**
1	Case Incidence	$\frac{Daily cases}{ total population}$ (per 100,000 population)	< 3	3 to < 7		7 to < 21		> = 21
2	Hospitalization rate	$\frac{Daily active cases}{ total population}$ (per 100,000 population)	< 5	5 to < 10		10 to < 30		> = 30
3	Mortality Rate	$\frac{Daily deaths}{Total population}$ (per 100,000 population)	< 1	1 to < 2		2 to < 5		5 & above
4	Effective Reproduction Number	$Rt=\frac{{I}_{t}}{\sum_{s=1}^{t}{I}_{t-s}{\omega }_{s}}$ *where I*_*t*_* is the number of infections incident on day t and ws is generation interval* [53]	< 0.5	0.5 to < 1		1 to < 1.3		> = 1.3
**RC level**								
**No**	**Indicators**	**Formula**	**Adequate**		**Moderate**		**Limited**	
1	Hospital bed capacity	$\frac{Daily active cases}{ total Hospital bed}$ (percentage)	< 75%		75% to < 90%		> = 90%	
2	COVID-19 ICU occupancy	No daily COVID-19 patients requiring ICU admission	< 60		60 to < 130		> = 130	
			< 700*		700 to < 1,200*		> = 1,200*	
3	Case fatality rate (COVID-19)	$\frac{Daily deaths}{Daily cases}$	Decrease (< 0)		stable (0)		Increase (> 0)	
4	Adherence to PHSM	Stringency index (0–100)	more than 70		30 to 70		< 30	
*Revised cut-off applied after February 2021 based on changes in COVID-19 hospitalization policy [54]

(b) Selecting CT indicator combinations

The indicators were combined to generate five combinations, wherein each combination have a common/constant indicator which was the case incidence as it accurately represented the disease transmission (Appendix 1). The CT values for each combination were measured by averaging individual CT values of each indicator as proposed by WHO as it produced the most accurate CT value [3].

(c) Validation of indicator combinations

Each CT indicator combination was validated using a correlation analysis $\left(r\right)$ between the daily CT values and observed COVID-19 cases from March 2020 to December 2020 which consisted of 283 days. The CT combination that generated the highest correlation was then selected as the most appropriate combination to generate CT levels for the pandemic. Before correlation analysis data was tested for normality using the Shapiro–Wilk test, wherein p-values were more than 0.05 indicating a non-normal distribution [32]. The Spearman’s rank-order correlation coefficient (ρ) was used to conduct the correlation analysis wherein ρ values between the ranges of less than 0.10, 0.10 to 0.39, 0.40 to 0.69, 0.70 to 0.89, and 0.90 to 1.00 indicated negligible, weak, moderate, strong, and very strong correlation, respectively [33]. The significance level of the correlation was set at p < 0.05 and Confidence Interval set at 95% [34].

1 $$r=1-\frac{6\sum {d}^{2}}{n({n}^{2}-1)}$$

where:

- ${r}_{s}$ is the Spearman's rank-order correlation coefficient
- $n$ is the number of pairs of data.
- ${d}_{i}$ is the difference between the ranks of corresponding variables in each pair of data.
  2 $$CI=\overline{x}\pm {t }_{\alpha /2}\times \frac{s}{\sqrt{n}}$$

Where:

- $\overline{x }$ is the sample mean.
- $s$ is the sample standard deviation.
- $n$ is the sample size.
- ${t}_{\alpha /2}$ is the critical value from the t-distribution with $n-1$ degrees of freedom, such that
- $P(-{t}_{\alpha /2}<T<{t}_{\alpha /2})=1-\alpha$, where a is the significance level (0.05 for a 95% Cl).
- $\pm$ represents the upper and lower bounds of the confidence interval.

#### Response capacity levels (RC)

The RC levels were estimated by determining the adequacy of response by the healthcare system during the COVID-19 pandemic. The RC values are derived from selecting the indicators such as clinical care capacity, clinical care performance, public health response capacity, and performance; and its cut-offs. The RC levels are categorized into adequate, moderate, or limited capacity [5].

- Selection of RC indicators and defining cuts off

The epidemiological indicators used to assess the RC level were hospital bed capacity, ICU occupancy, CFR, and adherence to PHSM as shown in Table 1. The WHO standard cut-offs were used to define the hospital bed capacity, CFR, and adherence to PHSM indicators. WHO standard cut-offs for ICU occupancy were revised after February 2021 based on changes in COVID-19 hospitalization policy to better represent the local situation in the respective RC levels [10] (Table 1). All cut-offs were validated during the validation process. Indicators were used individually and in combinations to determine the most appropriate RC values. Case incidence was used as a common indicator for each combination of indicators, therefore resulting in a total of 5 combinations as shown in Appendix 1.

(b) Selecting RC indicator combinations

The indicators were combined to generate five combinations, wherein each combination have a common/constant indicator which was the hospital bed capacity as it accurately represented the health system capacity (Appendix 1). The RC values for each combination were measured by averaging the individual RC values of each indicator as proposed by WHO as it produced the most accurate RC value [3].

(c) Validation of combinations

All RC combinations were validated using a correlation analysis between the daily RC values and observed COVID-19 cases from March 2020 to December 2020 which consisted of 283 days in a similar process as described above.

#### Determine and validate the COVID-19 Situational Level assessment matrix using the CT and RC variable

The COVID-19 Situational level assessment matrix is a composite variable that was determined by combining the validated CT and RC variable levels based on the WHO Situational Level matrix (Table 2), which ranges from no known transmission (Situational level 0) to uncontrolled epidemic (Situational level 4). Each situational level guides the required adjustment of the PHSMs. The determined COVID-19 Situational level assessment matrix was validated in the following ways:

**Table 2:**
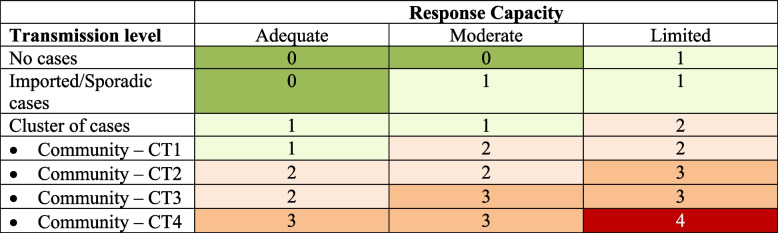
Situational level assessment matrix using transmission level and response capacity indicators to guide adjustment of PHSMs (Source WHO)

lower-alpha Correlation analysis between SL and daily COVID-19 cases

Data were tested for normality before the correlation analysis using the Shapiro–Wilk test, wherein *p* values < 0.05 indicate a normal distribution [8]. A correlation analysis between the daily Situational level values and observed daily COVID-19 cases from April 2020 to December 2020 which consisted of 275 days was performed using Spearman’s rank-order correlation coefficient (ρ) as mentioned above.

(b) Changes based on case trends

**Fig. 1:**
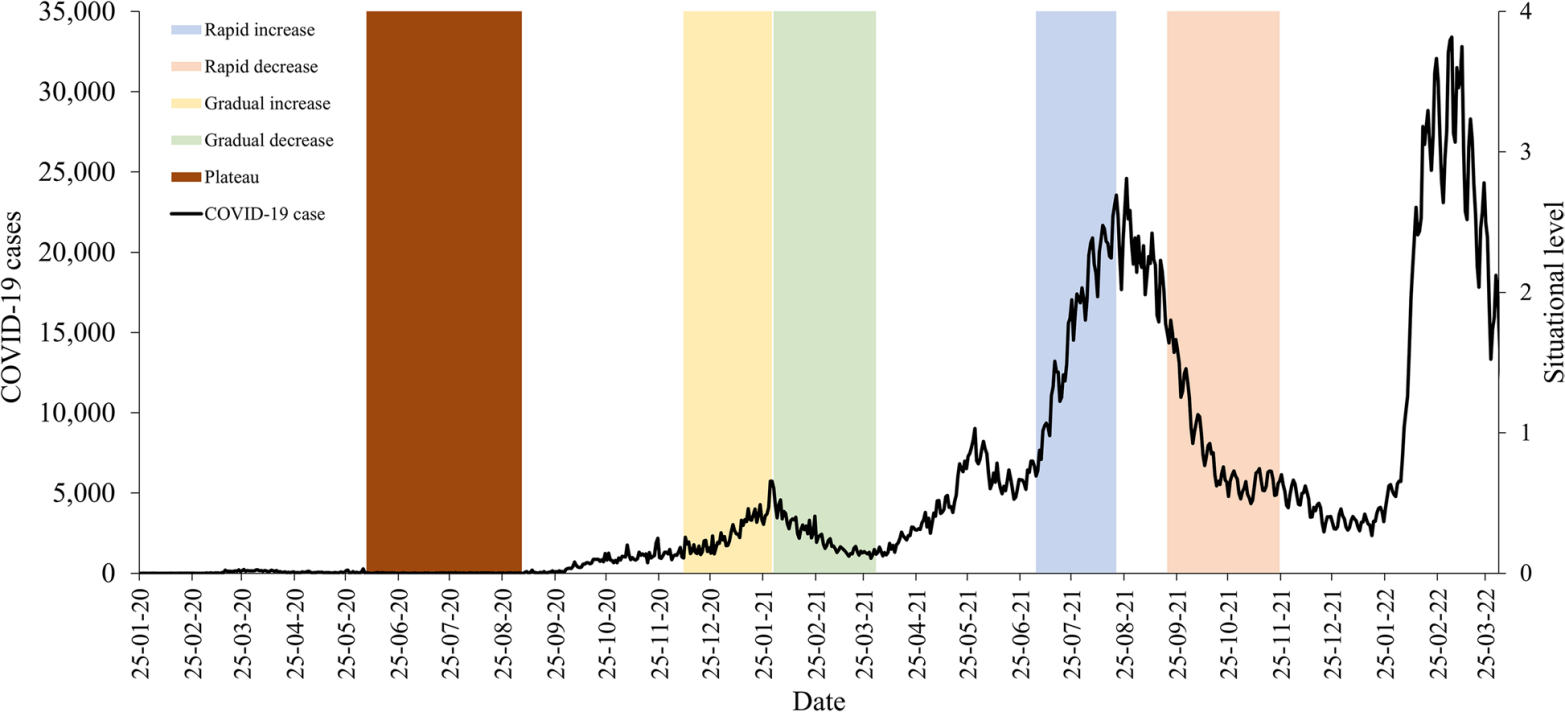
Trends of COVID-19 cases based on rapid increase/decrease, gradual increase/decrease, and plateau, Malaysia, 2020 – 2022

Changes in daily Situational level values in response to various COVID-19 case trends were examined and compared throughout the study period reflecting various scenarios. This is to determine the ability of the Situational level values to respond promptly to the changes in the case trends. Time series plots were developed to visualize the change in the Situational levels in response to various observed COVID-19 scenarios as follows; rapidly increasing trend (4/7/2021 to 20/8/2021), rapidly decreasing trend (19/9/2021 to 24/11/2021), gradually increasing trends (9/12/2020 to 30/1/2021), decreasing trend (31/1/2021 to 1/4/2021) and plateau trends (6/6/2020 to 5/9/2020) as shown in Fig. 1.

#### Developing an automated web-based COVID-19 Situational Level assessment matrix surveillance system

To provide daily COVID-19 Situational level assessment matrix estimates, an algorithm was developed and automated in the form of a web-based application using the R programming language with packages such as shiny, dplyr, dygraphs and ggplot [35–39]. This application extracts the COVID-19 case data for indicators estimation from relevant sources such as the MOH COVID-19 GitHub repository and DOSM portals. Automated parsing and structuring of extracted data were done in shiny server-side backend processing. Sourced data from the MOH COVID-19 GitHub repository and DOSM portals were transformed into a time series format using the incidence and xts packages of R, and the reactive function provided by R’s shiny application allows for reactive computation of outcomes based on user input. Data syncing and back-end computations were done to ensure the stability and ease of use of this application. This automated process updates the real-time daily COVID-19 Situational level assessment matrix and minimizes inconsistencies due to human error.

The schedule and automation function from the R programming language enables immediate updates as soon as required data are available and computes the COVID-19 Situational level assessment matrix values resulting in real-time COVID-19 Situational level assessment matrix updates. This process begins with the extraction of data from the MOH COVID-19 GitHub repository and reformats the data into an R-readable format that is compatible with the algorithm with functions from base R and dplyr. Pre-processed data which includes COVID-19 cases, recovered cases, death, hospitalized case, and ICU admission case count were then fitted into the system for calculation of the COVID-19 Situational level assessment matrix. An interactive interface with visual elements was also incorporated into the application to allow for smooth page rendering.

These automated and reactivity functions were developed in this application to provide the most optimal user experience. To ensure the application produces continuous daily COVID-19 Situational level assessment matrix outputs and to prevent downtime, several measures were taken. Firstly, downtime was prevented by introducing redundancies through hosting the application on an in-house primary and backup local server with uninterrupted power supply (UPS) protection as well as a backup power generation solution. Secondly, access to COVID-19 data was sourced from multiple open-access data sources in the event of data inaccessibility at the primary data source. Thirdly, the computational processes of the application can be overridden manually as a final failsafe mechanism in the event of automation failure. The automated web application system generates the daily COVID-19 Situational level assessment matrix flow using a back-end and a front-end process. This system enables users to monitor the daily COVID-19 Situational level assessment matrix values along with the CT, RC, and COVID-19 case trends in interactive plots at the national level.

### Data analysis software

Data was analyzed using the R programming software version 4.0.3 by Hornik and RCore Team, RStudio, PBC from 250 Northern Ave, Boston, MA, United States of America. Data pre-processing includes checking for missing data, normality tests, and transforming the data to a time series format. Time series graphs were developed using the Microsoft Corporation. (2018). Microsoft Excel. Retrieved from https://office.microsoft.com/excel. Data was presented in tabular and time series graphs.

## Results

### Selection and validation of the indicators to estimate the CT and RC levels

#### CT levels

Correlations (ρ) between individual indicators with COVID-19 cases ranged from 0.410 to 0.740. Of the five combinations the correlations (ρ) ranged from 0.451 to 0.786 (Table 3). Combination 1 which consisted of case incidence and hospitalization rate indicators produced the most appropriate CT levels compared to the other combinations, where the estimated CT values generated from combination 1 were strongly correlated (*ρ* = 0.806, *p* < 0.001) with the COVID-19 cases during the validation period (Table 3).

**Table 3:** Correlation between estimated CT, and RC levels with the daily COVID-19 cases in Malaysia, 2020

Indicator combination	Community Transmission	95% CI	*p*-value	Response Capacity	95% CI	*p*-value
1	0.806	0.752, 0.848	< 0.001	0.814	0.778, 0.839	< 0.001
2	0.666	0.584, 0.725	< 0.001	0.013	0.005, 0.001	0.826
3	0.451	0.345, 0.541	< 0.001	0.326	0.298, 0.351	< 0.001
4	0.786	0.736, 0.826	< 0.001	0.736	0.683, 0.784	< 0.001
5	0.746	0.677, 0.802	< 0.001	0.099	0.022, 0.175	0.095

*CI* Confidence Interval

#### RC levels

Correlations (ρ) between individual indicators with COVID-19 cases ranged from 0.444. to 0.545. Of the five combinations the correlations (ρ) ranged from 0.013 to 0.736 (Table 3). Combination 1 which consisted of hospital bed capacity and COVID-19 ICU occupancy indicators produced the most appropriate RC levels compared to the other combinations, where the estimated RC values generated from combination 1 were strongly correlated (*ρ* = 0.814, *p* < 0.001) with the COVID-19 cases during the validation period (Table 3).

### COVID-19 Situational level assessment matrix

**Fig. 2:**
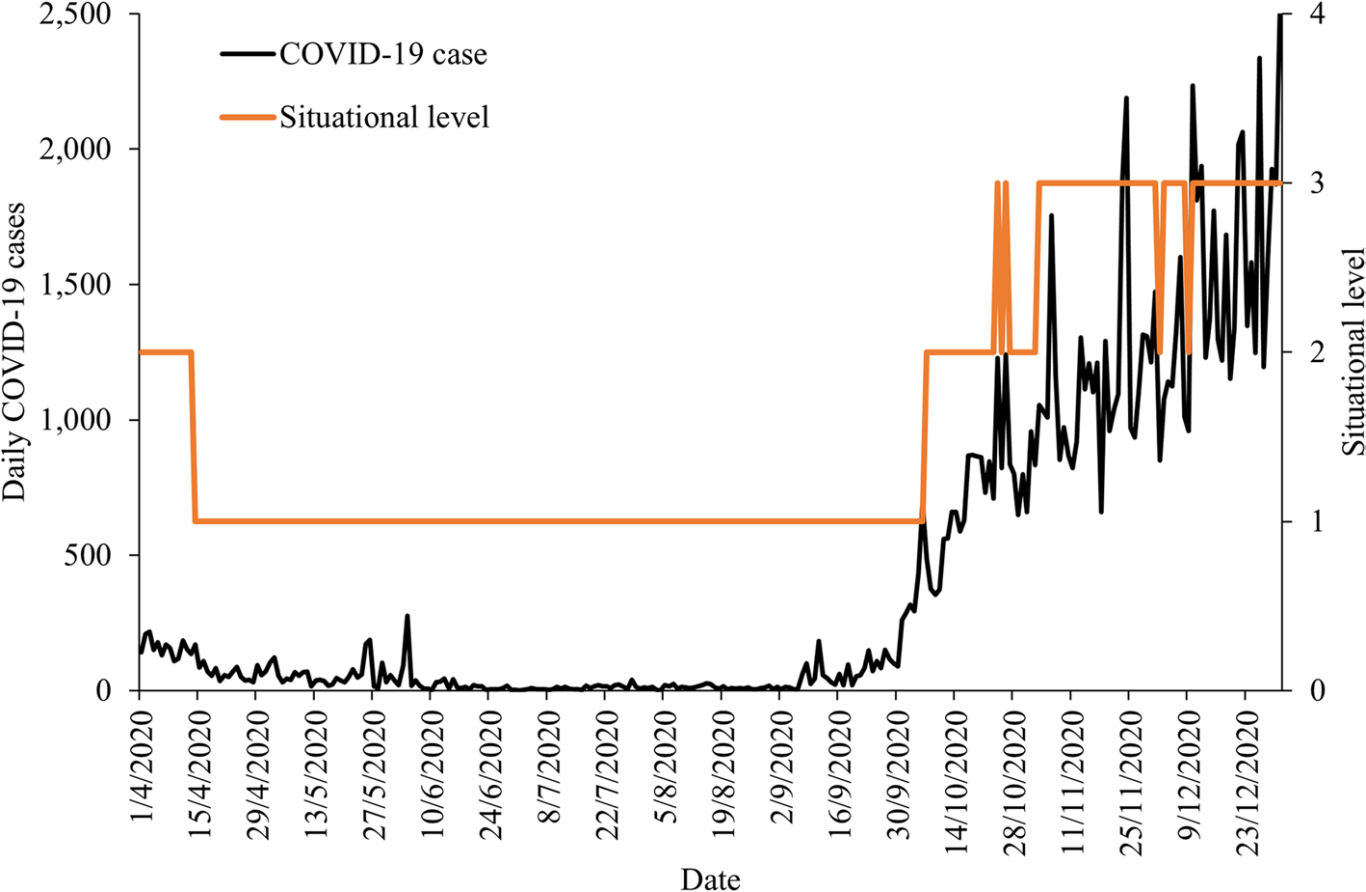
Correlation between estimated SL levels with the daily COVID-19 cases in, Malaysia, 2020

CT which is made from Case incidence and Hospitalization rate indicators; and RC which is Hospital bed capacity and COVID-19 ICU occupancy indicator generated the COVID-19 Situational level. The estimated COVID-19 Situational level assessment matrix levels were formed using the validated CT and RC value generated from combination 1 indicators respectively. The estimated Situational level was strongly correlated with COVID-19 cases (*ρ* = 0.845, *p* < 0.001) as shown in Fig. 2.

**Fig. 3:**
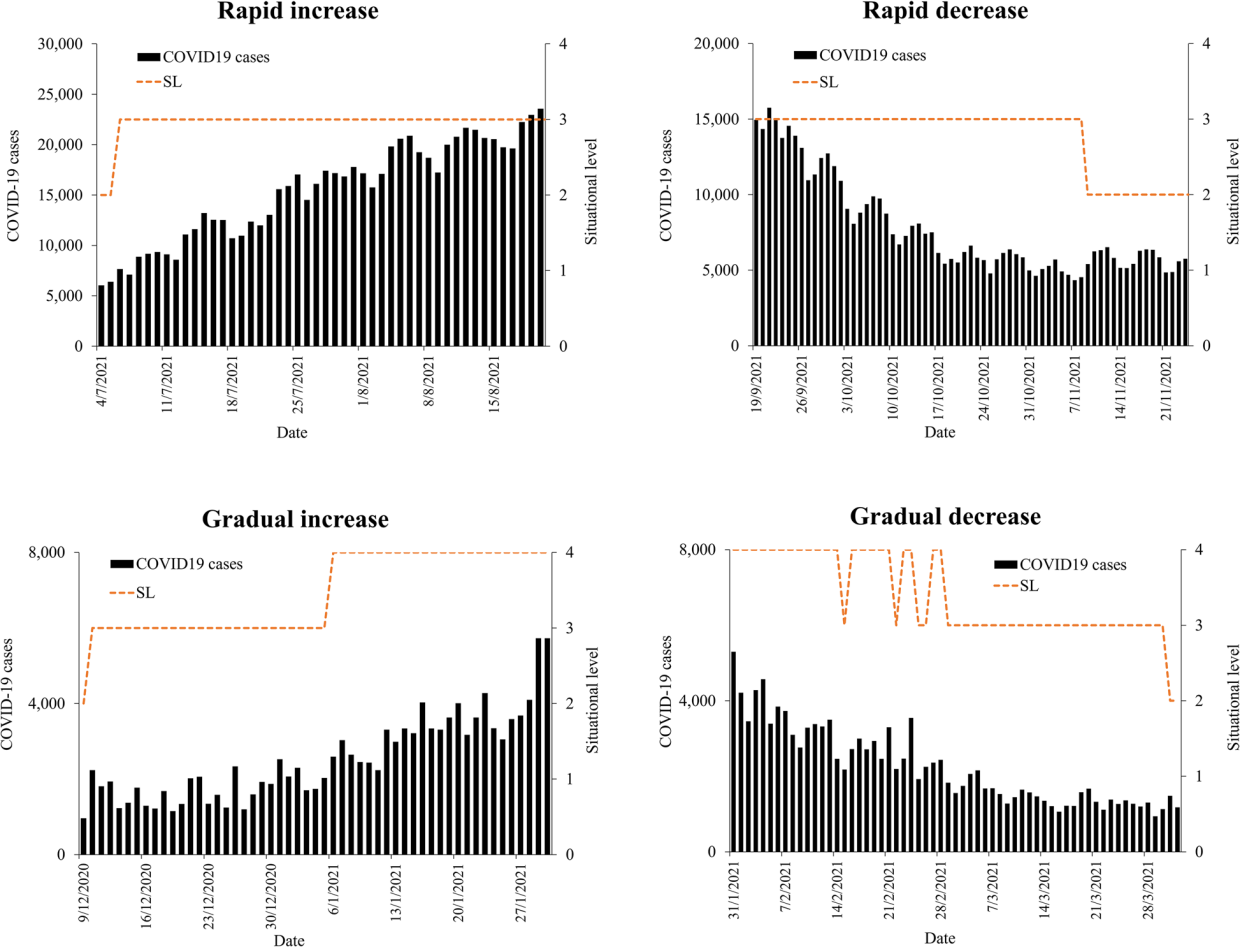
Changes in Situational levels following rapid increase/decrease and gradual increase/decrease in COVID-19 case trends, Malaysia, 2020 to 2021

**Fig. 4:**
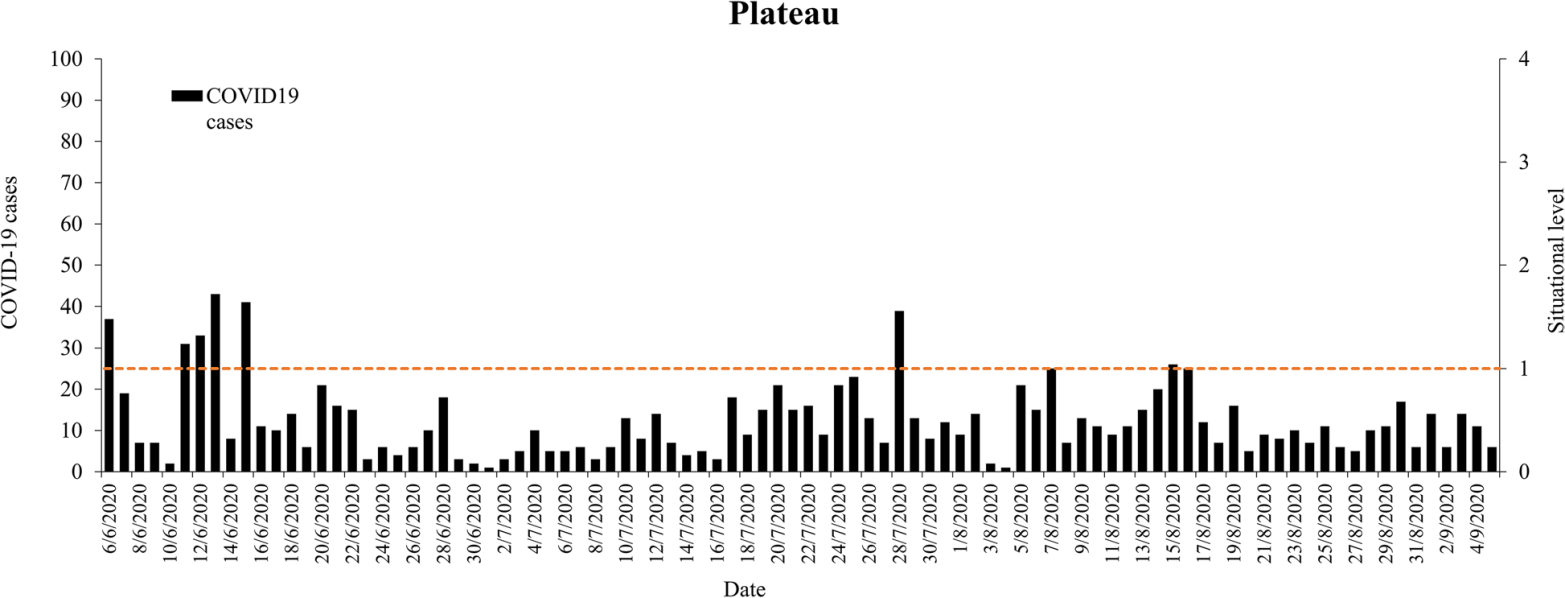
Changes in Situational levels following gradual increase/decrease and plateau COVID-19 case trends, Malaysia, 2020

In addition, the estimated Situational level responded well to the various COVID-19 selected scenarios (as shown in Fig. 3). Through the different scenarios (rapid/gradual increase/decrease/plateau) it was observed that the changes in the situational level corresponded closely to the changes in the case trends (Figs. 3 and 4). In addition, the changes in SL occurred earlier during rapid increase while the change in SL occurred slower during the rapid decrease which is indicative of a conservative response.

### Automated web-based COVID-19 Situational Level assessment matrix surveillance system

**Fig. 5:**
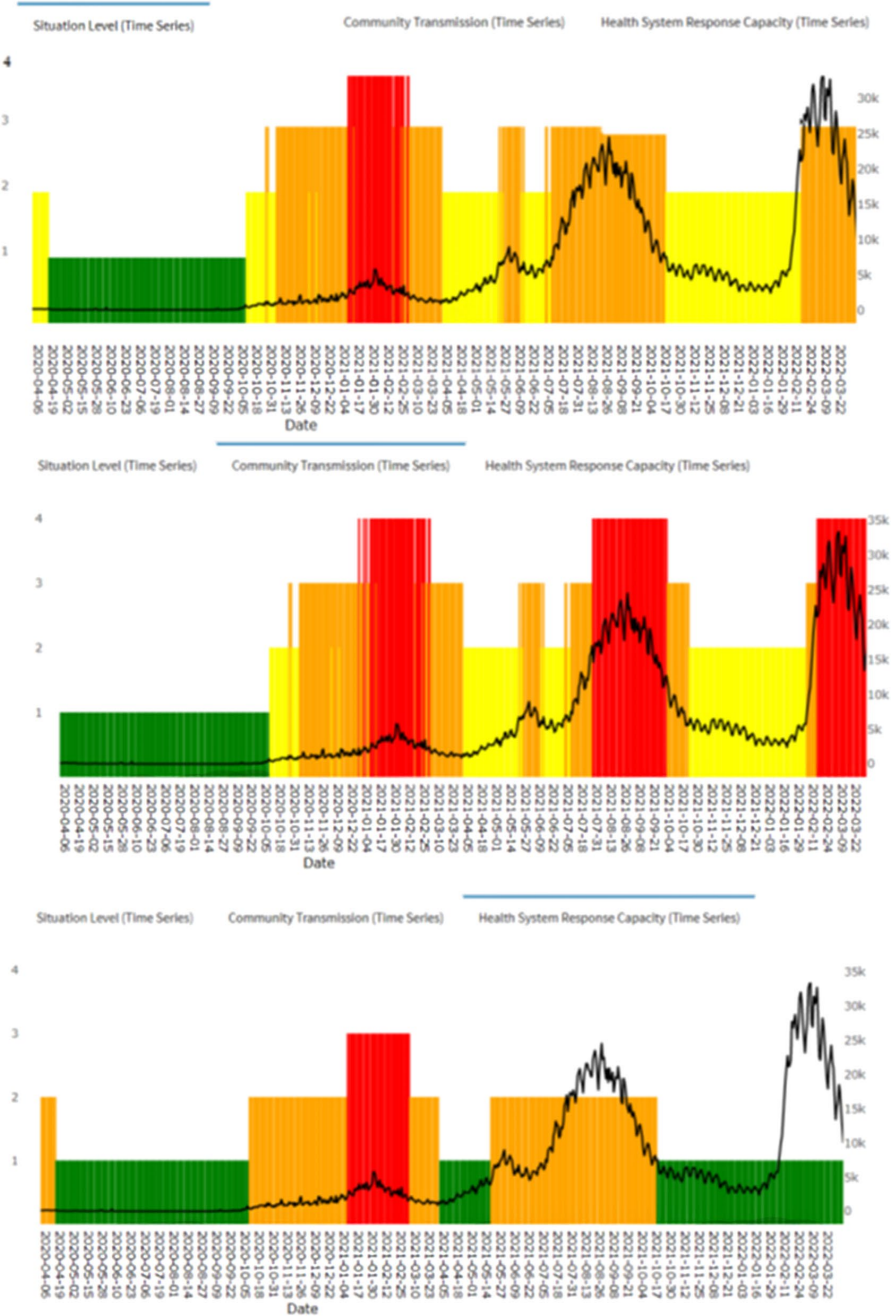
Automated web-based COVID-19 Situational Level assessment matrix surveillance system, Malaysia 2020 to 2022

The automated web-based COVID-19 Situational level surveillance system provides interactive time series plots which enable the user to get access to daily continuous real-time Situational, CT, and RC levels at the national level which are presented in the paragraphs below (Fig. 5). In addition, the system also displays the daily COVID-19 case which is superimposed on the CT, RC, and SL.

For the COVID-19 Situational level assessment matrix, during the initial phase of the COVID-19 pandemic in Malaysia, the Situational level was estimated at level 2, from 24/3/2020 to 13/4/2020, which corresponded to a duration of 21 days. Following this, the Situational level had reduced to level 1 and remained at this level for 176 days till 6/10/2020. Subsequently, the Situational level assessment consistently increased over the next few months from level 1 on 6/10/2020 to reach level 4 by 6/1/2021. Following this upward trend, the Situational level had reduced from level 4 to level 3 on 1/3/2021 and remained between levels 2 and 3 till the end of the study period. Through the study duration, the highest peak of the Situational level was recorded at level 4 which lasted for 54 days from 6/1/2021 to 28/2/2021 (Fig. 4).

During the initial phase of the pandemic, the CT levels had remained at level 1 and started gradually rising towards the end of 2020 to reach level 4 by January 2021. Subsequently, the CT levels fluctuated between levels 2, 3, and 4 till the end of the study period. Through the study period, the CT levels peaked at level 4 three times in total, twice in the year 2021 and once in 2022. Each CT level peak corresponded to the peak of COVID-19 cases during the respective periods (Fig. 4).

During the initial phase of the pandemic, the RC levels remained adequate and started gradually rising towards the end of 2020 to reach limited RC by January 2021. Subsequently, the RC levels fluctuated between adequate to moderate RC till finally being adequate RC from October 2021 onwards till the end of the study period. Through the study period, the RC levels peaked only once (limited RC) in early 2021 (Fig. 4).

## Discussion

The COVID-19 situational level (SL) reflects the extent of disease community transmission (CT) alongside the available health system response capacity resources (RC) to deal with the COVID-19 pandemic. In this study both the CT and RC levels were used to determine the SL, which is important because relying individually on levels of CT and RC alone is not sufficient and may be biased to determine the appropriate SL. For example, high CT levels may depict a poor SL, however, if the RC levels are adequate and both the CT and RC are accounted for together when assessing the SL then this would result in a better SL. The inverse is also true. Therefore, it is important to assess the SL by combining both the CT and RC levels as reported in this study [40, 41].

In this study, several indicators were used to determine the CT and RC levels. This is an important measure as the use of several indicators would allow for the formation of a more accurate and reflective SL, mainly because using multiple indicators would increase the variability and representativeness potential of the CT and RC level. Hence, identifying the relevant indicators to generate the most accurate CT and RC levels is an essential part of this study. Infection alone will not truly reflect CT if it's determined using only case counts, therefore, it is import include other indicators to provide a comprehensive reflection of CT. In this study, we report that the use of both COVID-19 case incidence and hospitalization rate indicators accurately estimated CT levels. This is because any changes in disease transmission would be reflected by the variation in number of infections and case incidence, wherein a person's risk for contracting the COVID-19 infection is directly related to the risk of exposure to an infectious person, which is largely determined by the extent of COVID-19 virus circulation in the surrounding community [42, 43]. Similarly, changes in disease transmission would affect the hospitalization rate (i.e. higher CT level would result in higher COVID-19 incidence and eventually more hospitalization) [44, 45]. While accurate RC levels were estimated in this study by using both the hospital bed capacity and COVID-19 ICU occupancy indicators. The use of these indicators was justified as RC is reflected by the ability of health systems to cope with the pandemic which is highly dependent upon the hospital bed capacity and COVID-19 ICU occupancy [46–48]. Any strains on hospital bed capacity and COVID-19 ICU occupancy would result in limited RC which ultimately could cause an overwhelm healthcare system and an increase in COVID-19 mortality [49–51]. Similarly, approaches have been used to assess the CT and RC levels in previous studies [49–52]. Where most of them concluded that a minimum of two indicators are required to produce reliable estimates for the CT and RC levels, provided that these indicators can adequately represent the disease transmission and healthcare capacity [49–52].

In this study, all the indicator was measured on a continuous scale hence it was important to ensure that the cut-off used for each indicator is appropriate as this in turn would determine the level classification. Therefore, to address this issue, several strategies were undertaken, first, the WHO standard cut-offs were applied to all indicators and subsequently were validated during the validation phase. WHO cut-offs that were valid were retained and used for the relevant indicators. A revised cut-off was used for the COVID-19 ICU occupancy indicator based on changes in COVID-19 hospitalization policy since February 2021 [31]. These strategies ensured that the cut-offs applied were suitable for the local context. In addition, all the indicators within a given combination were averaged which resulted in the generation of better CT and RC levels. This is because using an average method equally accounts for the effects of individual indicators within a given combination instead of relying only on the worst or top priority indicator values which would bias the overall estimation. Furthermore, in this study, several validation processes were performed which included validation of the indicators selected to form the CT and RC levels, validation of the cut-off applied to these indicators, and validation of the CT, RC, and SL levels. The validation process ensured that the indicators selected alongside the cut-off applied were appropriate to form reasonably accurate CT and RC levels as evidenced by the strong significant correlations between the estimated CT, RC, and SL levels with the daily COVID-19 cases. As a result, the SL corresponded well to the changes in various COVID-19 case trends (i.e. gradual increase/decrease and plateau) during the pandemic in Malaysia as reported in this study. These findings support the validity of the CT, RC, and SL estimates generated in this study. Moreover, the prevailing SL estimates produced in this study exhibit a similar trend to the Oxford Stringency Index for Malaysia, albeit with some nuanced discrepancies that may be elucidated by variations in the indicators employed.

The daily Situational level matrix for Malaysia was developed by combining the CT and RC levels to form a composite matrix using the WHO framework [5]. While most system relies on a single-based measure (i.e. response capacity or disease transmission) the SL generated in this study is more representative as it accounts for both the CT and RC levels. The estimated daily Situational level was reported to be valid as it was strongly correlated with the COVID-19 cases. In addition, the changes in the Situational level corresponded well with the changes in the observed case trends. Where the Situational level increased quickly as a response to the rapidly rising case trends. However, a gradual reduction in Situational is observed following a rapid decline in cases. Also despite the rise in cases, the SL may not necessarily increase to its highest level. This is because the Situational level matrix factors in both the CT and RC components, therefore changes in case of trend only without a change in active case (response capacity) would not trigger a change in the Situational level. This is a useful finding as the early rise in SL would allow for the early institution of pandemic control measures while the gradual reduction in SL despite reductions of COVID-19 cases would ensure a more cautious approach in adjusting pandemic control measures. Taken together this would prevent the occurrence of severe outbreaks or resurgence of cases. This in all provides evidence that the Situational level matrix estimated in this study is not only responsive to the changes in case trends but also conservative and cautious when deciding on changing its levels. This is important to ensure appropriate adjustment of PHSM while avoiding premature lifting/relaxation which could result in a resurgence of cases. The SL generated in this study provided a holistic representation of the COVID-19 pandemic progression in Malaysia. When there is evidence of rising CT levels and inadequate health system capacity there is an urgent need to step up the available PHSM. This measure would temporarily suppress the transmission of COVID-19 infections, therefore reducing daily COVID-19 cases and possibly preventing an uncontrolled epidemic that may overwhelm the health system resulting in high SL (i.e. Level 4) [5].

To date, no system in place provides daily CT, RC levels, and Situational levels for the COVID-19 pandemic in Malaysia. The availability of real-time information on daily CT, RC, and Situational levels generated by the automated web-based system in this study could be used as a surveillance tool to monitor the progress of the pandemic in Malaysia and provide direction on whether containment measures (lower situational level) or mitigation measures (higher situational level) should be adopted. Furthermore, continuous data on the Situational level would enable the government to plan and mobilize resources effectively to ensure that there are sufficient treatment facilities, medical equipment, and healthcare workers to cope with surges and possible resurges of COVID-19 infections, and enable health authorities to consider implementing and adjusting various PHSM specific to each situation level as suggested by WHO, while objectively assessing and monitoring the effects of the PHSM on disease transmission and health system capacity levels [5]. Therefore, the availability of a comprehensive COVID-19 Situational level system in place as developed in this study would surely supplement the existing COVID-19 control and management strategies in Malaysia.

There are several strengths to the current work, first is the use of both CT and RC to provide a reasonable SL estimate. Also the use of more than one indicator to estimate the CT and RC increases the variability and representativeness potential of the CT and RC level. The CT, RC, and SL were found to be valid as they correlated well with the COVID-19 case trends. In addition, the SL generated in this study adopts a conservative approach which is more suited to prevent the rebound of infections due to the premature release of PHSM. Moreover, the automated web-based system can generate real-time daily SL estimates which could be a useful tool for disease surveillance. Among the limitation of this study include the representation of the indicators used to estimate CT and RC wherein we identified 2 suitable indicators out of the 5 indicators proposed by WHO. Nevertheless, the CT and RC level estimated using the combination of these 2 indicators were found to be valid and reliable in this study. The cut-off used in the classification of the indicators where based on the WHO cut off and some were adjusted to suit the current local context during the study period however it may require revision from time to time to be able to account for the changes in disease dynamics over time. Nevertheless, this study was able to produce CT, RC, and Situational level matrix which is accurate and reliable.

## Conclusion

In the context of combating a novel and rapidly evolving disease such as COVID-19, the utilization of Public Health and Social Measures (PHSM) becomes not only essential but paramount. This strategy requires a delicate balancing act, ensuring the protection of public health while simultaneously safeguarding livelihoods. Achieving this equilibrium necessitates a comprehensive approach that takes into account a myriad of indicators to make well-informed decisions.

Beyond solely relying on the widely used community transmission (CT) indicator, it is imperative to broaden our scope to incorporate the assessment of health response capacity (RC). The RC acts as a critical barometer, signaling potential strains on the healthcare system that could have far-reaching consequences. Through the meticulous incorporation of both CT and RC, validated and proven effective in this study, we can construct a robust COVID-19 Situational Level Matrix. This approach is characterized by its conservative nature, ensuring a cautious yet effective control of disease transmission.

Furthermore, the findings of this study extend beyond the confines of the current COVID-19 pandemic, offering valuable insights applicable to various disease outbreaks. This underscores the versatility and adaptability of our approach in addressing emerging public health challenges.

In summary, this study underscores the critical importance of utilizing multiple indicators, including CT and RC, to inform evidence-based decision-making in managing disease outbreaks. By striking a delicate balance between proactive measures and continuous vigilance, we can effectively mitigate the burden of diseases and safeguard public health, thereby ensuring the well-being of communities both now and in the future.

## Data Availability

No datasets were generated or analysed during the current study.

## Appendix

### Indicator combinations used to develop CT and RC

**Table:**

Indicator combination	Community Transmission	Response Capacity
1	Case incidence	Hospital bed capacity
	Hospitalization rate	COVID-19 ICU occupancy
2	Case incidence	Hospital bed capacity
	Mortality rate	CFR
3	Case incidence	Hospital bed capacity
	Effective reproduction number	Adhere to PHSM
4	Case incidence	Hospital bed capacity
	Hospitalization rate	COVID-19 ICU occupancy
	Mortality rate	CFR
5	Case incidence	Hospital bed capacity
	Hospitalization rate	COVID-19 ICU occupancy
	Mortality rate	CFR
	Effective reproduction number	Adhere to PHSM

## References

[1] World Health Organization. Archived: WHO timeline - COVID-19. 2020. Available from: https://www.who.int/news/item/27-04-2020-who-timeline-covid-19. Accessed 1 Jan 2021.

[2] Naseer S, Khalid S, Parveen S, Abbass K, Song H, Achim MV (2022). COVID-19 outbreak: impact on global economy. Front Public Health.

[3] Han E, Tan MMJ, Turk E, Sridhar D, Leung GM, Shibuya K (2020). Lessons learnt from easing COVID-19 restrictions: an analysis of countries and regions in Asia Pacific and Europe. Lancet.

[4] Rehfuess EA, Movsisyan A, Pfadenhauer LM, Burns J, Ludolph R, Michie S (2023). Public health and social measures during health emergencies such as the COVID-19 pandemic: an initial framework to conceptualize and classify measures. Influenza Other Respir Viruses.

[5] World Health Organization. Considerations in adjusting public health and social measures in the context of COVID-19,14 June 2021. WHO Press; 2021. p. 1–13. Available from: https://www.paho.org/en/documents/considerations-implementing-and-adjusting-public-health-and-social-measures-context-covid. Cited 2021 Sep 25.

[6] Lovelace B Jr . CNBC; Englewood Cliffs. 2020. WHO: countries that rush to lift restrictions risk ‘severe and prolonged’ damage to economy. https://www.cnbc.com/2020/04/03/who-says-countries-that-rush-to-lift-coronavirus-containment-risk-more-severe-and-prolonged-damage-to-economy.html.

[7] Tang KHD. Movement control as an effective measure against Covid-19 spread in Malaysia: an overview. Z Gesundh Wiss. 2022;30(3):583–6. 10.1007/s10389-020-01316-w. Epub 2020 Jun 13. 10.1007/s10389-020-01316-w PMC7293423 32837842

[8] Herng LC, Singh S, Sundram BM, Zamri ASSM, Vei TC, Aris T (2022). The effects of super spreading events and movement control measures on the COVID-19 pandemic in Malaysia. Sci Rep.

[9] Ganasegeran K, Ch'ng ASH, Looi I (2020). COVID-19 in Malaysia: crucial measures in critical times. J Glob Health.

[10] Adam D (2020). Special report: the simulations driving the world’s response to COVID-19. Nature.

[11] Zhang J, Litvinova M, Wang W, Wang Y, Deng X, Chen X (2020). Evolving epidemiology and transmission dynamics of coronavirus disease 2019 outside Hubei province, China: a descriptive and modelling study. Lancet Infect Dis.

[12] Chinazzi M, Davis JT, Ajelli M, Gioannini C, Litvinova M, Merler S (2020). The effect of travel restrictions on the spread of the 2019 novel coronavirus (COVID-19) outbreak. Science.

[13] Gale D (1955). The law of supply and demand. Math Scand.

[14] Guan J, Zhao Y, Wei Y, Shen S, You D, Zhang R, et al. Transmission dynamics model and the coronavirus disease 2019 epidemic: applications and challenges. 2022;2(1):89–109. 10.1515/mr-2021-0022. 10.1515/mr-2021-0022 PMC9047651 35658113

[15] Kruk ME, Gage AD, Arsenault C, Jordan K, Leslie HH, Roder-DeWan S (2018). High-quality health systems in the sustainable development goals era: time for a revolution. Lancet Glob Heal.

[16] Kieny MP, Evans DB, Schmets G, Kadandale S (2014). Health-system resilience: reflections on the Ebola crisis in western Africa. Bull World Health Organ.

[17] Leech G, Rogers-Smith C, Monrad JT, Sandbrink JB, Snodin B, Zinkov R (2022). Mask wearing in community settings reduces SARS-CoV-2 transmission. Proc Natl Acad Sci U S A.

[18] Sun K, Wang W, Gao L, Wang Y, Luo K, Ren L (2021). Transmission heterogeneities, kinetics, and controllability of SARS-CoV-2. Science.

[19] Prem K, Liu Y, Russell TW, Kucharski AJ, Eggo RM, Davies N (2020). The effect of control strategies to reduce social mixing on outcomes of the COVID-19 epidemic in Wuhan, China: a modelling study. Lancet Public Heal.

[20] Chu DK, Akl EA, Duda S, Solo K, Yaacoub S, Schünemann HJ (2020). Physical distancing, face masks, and eye protection to prevent person-to-person transmission of SARS-CoV-2 and COVID-19: a systematic review and meta-analysis. Lancet.

[21] Islam N, Sharp SJ, Chowell G, Shabnam S, Kawachi I, Lacey B (2020). Physical distancing interventions and incidence of coronavirus disease 2019: natural experiment in 149 countries. BMJ.

[22] Marome W, Shaw R (2021). COVID-19 response in Thailand and its implications on future preparedness. Int J Environ Res Public Health.

[23] Hale T, Angrist N, Goldszmidt R, Kira B, Petherick A, Phillips T (2021). A global panel database of pandemic policies (Oxford COVID-19 Government Response Tracker). Nat Hum Behav.

[24] Tan CS, Lokman S, Rao Y, Kok SH, Ming LC (2021). Public and private sectors collective response to combat COVID-19 in Malaysia. J Pharm Policy Pract.

[25] Muhammad Nur Amir AR, Binti Amer Nordin A, Lim YC, Binti Ahmad Shauki NI, Binti Ibrahim NH (2021). Workforce mobilization from the National Institutes of Health for the Ministry of Health Malaysia: a COVID-19 pandemic response. Front public Heal.

[26] Md Hamzah N, Yu MM, See KF (2021). Assessing the efficiency of Malaysia health system in COVID-19 prevention and treatment response. Health Care Manag Sci.

[27] Rader B, Scarpino SV, Nande A, Hill AL, Adlam B, Reiner RC (2020). Crowding and the shape of COVID-19 epidemics. Nat Med.

[28] Eubank S, Eckstrand I, Lewis B, Venkatramanan S, Marathe M, Barrett CL, Commentary on Ferguson (2020). Impact of Non-pharmaceutical Interventions (NPIs) to Reduce COVID-19 Mortality and Healthcare Demand. Bull Math Biol..

[29] Menzies NA, Gomez GB, Bozzani F, Chatterjee S, Foster N, Baena IG (2016). Cost-effectiveness and resource implications of aggressive action on tuberculosis in China, India, and South Africa: a combined analysis of nine models. Lancet Glob Heal.

[30] Stutt ROJH, Retkute R, Bradley M, Gilligan CA, Colvin J (2020). A modelling framework to assess the likely effectiveness of facemasks in combination with “lock-down” in managing the COVID-19 pandemic. Proc Math Phys Eng Sci.

[31] MOH. COVID-19 press statement-from the desk of the director-general of health Malaysia. MOH official website. 2022. Available from: https://kpkesihatan.com/2022/04/01/kenyataan-akhbar-kpk-1-april-2022-situasi-semasa-jangkitan-penyakit-coronavirus-2019-covid-19/. Cited 2022 Jun 29.

[32] Jurečková J, Picek J (2007). Shapiro-Wilk-type test of normality under nuisance regression and scale. Comput Stat Data Anal.

[33] Schober P, Schwarte LA (2018). Correlation coefficients: appropriate use and interpretation. Anesth Analg.

[34] Altman DG, Bland JM (2005). Standard deviations and standard errors. BMJ.

[35] R Core Team. R: A language and environment for statistical computing. R Foundation for Statistical Computing. 2019. Available from: https://www.gbif.org/tool/81287/r-a-language-and-environment-for-statistical-computing. Accessed 1 Jan 2021.

[36] Chang W, Cheng J, Allaire J, Sievert C, Schloerke B, Xie Y, et al. Shiny: web application framework for R. R package version 1.7.1. 2021. Available from: https://cran.r-project.org/package=shiny. Accessed 1 Jan 2021.

[37] Wickham H, François R, Henry L, Müller K. Dplyr: a grammar of data manipulation. R package version 1.0.9. 2022. Available from: https://cran.r-project.org/package=dplyr. Accessed 1 Jan 2021.

[38] Vanderkam D, Allaire J, Owen J, Gromer D, Thieurmel B. Dygraphs: interface to “Dygraphs” interactive time series charting library. R package version 1.1.1.6. 2018. Available from: https://cran.r-project.org/package=dygraphs. Accessed 1 Jan 2021.

[39] Wickham H (2016). ggplot2: elegant graphics for data analysis.

[40] Davoudi B, Moser F, Brauer F, Pourbohloul B (2013). Epidemic progression on networks based on disease generation time. J Biol Dyn.

[41] Kandel N, Chungong S, Omaar A, Xing J (2020). Health security capacities in the context of COVID-19 outbreak: an analysis of International Health Regulations annual report data from 182 countries. Lancet.

[42] Dabholkar YG, Sagane BA, Dabholkar TY, Divity S, Cheng C, Wan X (2020). Modeling analysis reveals the transmission trend of COVID-19 and control efficiency of human intervention. BMC Infect Dis.

[43] Dabholkar YG, Sagane BA, Dabholkar TY, Divity S (2020). COVID19 infection in health care professionals: risks, work-safety and psychological issues. Indian J Otolaryngol Head Neck Surg.

[44] Todd IMF, Miller JE, Rowe SL, Burgner DP, Sullivan SG (2021). Changes in infection-related hospitalizations in children following pandemic restrictions: an interrupted time-series analysis of total population data. Int J Epidemiol.

[45] Cassell K, Zipfel CM, Bansal S, Weinberger DM (2022). Trends in non-COVID-19 hospitalizations prior to and during the COVID-19 pandemic period, United States, 2017–2021. Nat Commun.

[46] El Bcheraoui C, Weishaar H, Pozo-Martin F, Hanefeld J (2020). Assessing COVID-19 through the lens of health systems’ preparedness: time for a change. Glob Health.

[47] Knutsen Glette M, Ludlow K, Wiig S, Bates DW, Austin EE (2023). Resilience perspective on healthcare professionals’ adaptations to changes and challenges resulting from the COVID-19 pandemic: a meta-synthesis. BMJ Open.

[48] Mohammadinia L, Saadatmand V, Khaledi Sardashti H, Darabi S, Esfandiary Bayat F, Rejeh N (2023). Hospital response challenges and strategies during COVID-19 pandemic: a qualitative study. Front Public Health.

[49] Whaley CM, Pera MF, Cantor J, Chang J, Velasco J, Hagg HK (2020). Changes in health services use among commercially insured US populations during the COVID-19 pandemic. JAMA Netw open.

[50] Bravata DM, Perkins AJ, Myers LJ, Arling G, Zhang Y, Zillich AJ (2021). Association of intensive care unit patient load and demand with mortality rates in US Department of Veterans affairs hospitals during the COVID-19 pandemic. JAMA Netw Open.

[51] Karaca-Mandic P, Sen S, Georgiou A, Zhu Y, Basu A. Association of COVID-19-Related Hospital Use and Overall COVID-19 Mortality in the USA. J Gen Intern Med. 2020:1–3. 10.1007/s11606-020-06084-7. Epub ahead of print. 10.1007/s11606-020-06084-7 PMC7437636 32815058

[52] A C, JT B, LA H. Guidance for implementing COVID-19 prevention strategies in the context of varying community transmission levels and vaccination coverage. MMWR Morb Mortal Wkly Rep. 2021. Available from: https://www.cdc.gov/mmwr/volumes/70/wr/mm7030e2.htm#suggestedcitation. Cited 2022 Aug 8. 10.15585/mmwr.mm7030e2 PMC8323553 34324480

[53] Gostic KM, McGough L, Baskerville EB, Abbott S, Joshi K, Tedijanto C, Kahn R, Niehus R, Hay JA, De Salazar PM, Hellewell J, Meakin S, Munday JD, Bosse NI, Sherrat K, Thompson RN, White LF, Huisman JS, Scire J, Bonhoeffer S, Stadler T, Wallinga J, Funk S, Lipsitch M, Cobey S. Practical considerations for measuring the effective reproductive number, Rt. PLoS Comput Biol. 2020;16(12):e1008409. 10.1371/journal.pcbi.1008409. Erratum in: PLoS Comput Biol. 2021;17(12):e1009679. 10.1371/journal.pcbi.1008409 PMC7728287 33301457

[54] Ministry of Health Malaysia. From the desk of the director-general of health Malaysia - Kenyataan Akhbar KPK 7 Februari 2022 – Situasi Semasa Jangkitan Penyakit Coronavirus 2019 (COVID-19). 2022. Available from: https://kpkesihatan.com/2022/02/07/kenyataan-akhbar-kpk-7-februari-2022-situasi-semasa-jangkitan-penyakit-coronavirus-2019-covid-19/. Cited 2022 Sep 23.

